# Preclinical Efficacy and Safety of an Oncolytic Adenovirus KD01 for the Treatment of Bladder Cancer

**DOI:** 10.3390/ph18040511

**Published:** 2025-03-31

**Authors:** Jin Guo, Shengfeng Xiong, Xinyuan Zhang, Wei Gong, Yao Si, Ding Ma, Fei Li, Yingyan Han

**Affiliations:** 1 Department of Obstetrics and Gynecology, Tongji Hospital, Tongji Medical College, Huazhong University of Science and Technology, Wuhan 430034, China; 2 National Clinical Research Centre for Obstetrics and Gynecology, Cancer Biology Research Centre (Key Laboratory of the Ministry of Education), Tongji Hospital, Tongji Medical College, Huazhong University of Science and Technology, Wuhan 430034, China; 3 Department of Neurosurgery, Tongji Hospital, Tongji Medical School, Huazhong University of Sciences and Technology, Wuhan 430030, China

**Keywords:** bladder cancer, KD01, oncolytic adenovirus, cisplatin, immunogenic cell death

## Abstract

**Background**: While Bacillus Calmette-Guérin (BCG) remains the first-line therapy for high-risk bladder cancer, 30–40% of patients develop treatment resistance necessitating radical cystectomy, some are not suitable candidates for this procedure. This underscores the critical need for novel therapeutic approaches. Emerging clinical evidence has increasingly supported the therapeutic potential of oncolytic viruses in bladder cancer treatment. Based on this clinical foundation, we investigated the anti-tumor effects of KD01, a novel type 5 recombinant oncolytic adenovirus previously developed by our team engineered to express truncated BID (tBID), in bladder cancer. **Methods**: The cytotoxic effects and anti-tumor efficacy of KD01 were systematically evaluated across human bladder cancer cell lines, and cell death pathways were investigated by RNA sequencing and validated. Combination therapy studies with cisplatin employed cytotoxic testing. In the final stage, the safety of KD01 bladder instillation was evaluated. **Results**: KD01 induced bladder cancer cell death through multiple mechanisms, including oncolysis, immunogenic cell death, and mitochondrial apoptosis. At higher doses, KD01 combined with cisplatin synergistically inhibited cancer cell proliferation and induced apoptosis. Additionally, KD01 amplified damage-associated molecular patterns (DAMPs) release and immune activation; the combination with cisplatin further enhanced the process. Safety evaluations showed favorable tolerance to intravesical perfusion with KD01. **Conclusions**: The dual action of KD01 in directly killing tumor cells and activating anti-tumor immunity underscores its potential as a therapeutic agent. These findings highlight the preclinical efficacy and safety of KD01, informing the design of clinical trials.

## 1. Introduction

Bladder cancer (BC) ranks as the fifth most prevalent malignancy worldwide, with an estimated annual incidence exceeding 573,000 new cases, and its incidence continues to rise [1,2]. Current classification stratifies BC into three principal categories: non-muscle-invasive bladder cancer (NMIBC), non-metastatic disease, muscle-invasive bladder cancer (MIBC), and metastatic bladder cancer [3]. While NMIBC accounts for 75–85% of initial diagnoses and exhibits 5-year survival rates exceeding 90%, its high recurrence rate (50–70%) and progression potential to MIBC (10–15%) necessitate vigilance [4,5]. Current intravesical agents include chemotherapeutics such as mitomycin C, epirubicin, gemcitabine, and Bacillus Calmette-Guérin (BCG) [6,7]. First-line intravesical therapy with Bacillus Calmette-Guérin (BCG) achieves complete responses in 55–68% of high-risk NMIBC patients, yet 30–40% develop a BCG-unresponsive disease requiring radical cystectomy (RC). Nonetheless, many patients either refuse or are unsuitable for RC [8,9]. Despite these interventions, the recurrence rates are significant, with 50–70% of patients experiencing relapse within five years, and 10–15% of NMIBC patients progressing to MIBC [10]. This therapeutic impasse underscores the urgent need for bladder-preserving alternatives.

The advent of oncolytic virotherapy has reshaped the bladder cancer treatment landscape. FDA approval of nadofaragene firadenovec (Adstiladrin), a non-replicating adenoviral vector delivering interferon-α2b, demonstrated 53.4% complete response rates at 3 months in BCG-unresponsive NMIBC [11,12]. Parallel developments include cretostimogene grenadenorepvec (CG0070), a GM-CSF-armed oncolytic adenovirus achieving 75.2% CR in phase III trials [13]. The achievements of the oncolytic virus underscore its potential as a therapeutic strategy for this challenging patient population, with better therapeutic efficacy than BCG; however, the CR rate is not a satisfactory one when used alone, and the combination places a greater burden on the patient. The oncolytic virus can be modified to carry some genes to increase efficiency further [14,15]. The adenovirus is one of the most researched viruses in the field of oncolytic virus therapy, and its oncolytic methods include the direct killing of tumor cells and activation of the body immune reaction to inhibit tumor development [16,17]. Recombinant oncolytic adenovirus (KD01) is a type 5 recombinant oncolytic adenovirus that carries the apoptosis gene tBID, which shows conditional replication ability. KD01 can conditionally replicate in ovarian cancer cells, and triggering apoptosis, cisplatin combination therapy improves anti-tumor efficacy in patients with advanced ovarian cancer [18,19,20]. Therefore, we performed preclinical pharmacodynamic evaluations across multiple solid tumor models, revealing that KD01 exhibits significant anti-tumor efficacy in bladder cancer. However, its potential synergistic effects with chemotherapy agents and the precise mechanisms underlying tumor cell death induction remain unclear.

Oncolytic virus monotherapy limitations persist, such as incomplete tumor clearance and insufficient immune activation. The standard of care for patients with MIBC is cystectomy accompanied by adjuvant chemotherapy, but not all patients are candidates for resection therapy. The concern in patients with MIBC is the metastasis of cancer and the difficulty in detecting and effectively treating the deep, tiny cancer foci, so pharmacologic systemic therapy has emerged as an alternative treatment modality. In a phase 2 trial, the combination of gemcitabine, cisplatin, and anti-PD-1 resulted in 43% of patients with MIBC in complete clinical remission [21], although the rate of complete clinical remission is not a significant one, it provides a promising option for patients who cannot undergo cystectomy. Cisplatin is a widely used chemotherapeutic agent that inhibits cell proliferation by inducing DNA damage, and its therapeutic mechanism also appears to be related to the promotion of apoptosis. This article investigates the therapeutic effect of KD01 in combination with cisplatin in bladder cancer, which adds an additional mode of use to the clinical use of KD01.

KD01 has received Investigational New Drug (IND) approval from the National Medical Products Administration (NMPA) and is currently undergoing Phase I clinical trials targeting over ten advanced solid tumors (Clinical Trial Acceptance Number: CXSL2300531). Our findings establish a pharmacodynamic foundation for ongoing investigator-initiated trials exploring intravesical KD01 in BCG-refractory NMIBC, while informing rational combination strategies for MIBC patients ineligible for cystectomy.

## 2. Results

### 2.1. KD01 Has a Killing Effect on Bladder Cancer Cells In Vitro

KD01 is a conditionally replicating oncolytic adenovirus (oAd) in which the E1A CR2 (920–946 nt) and E3 ADP regions have been deleted and replaced with the pro-apoptotic gene *tBID*. It has received approval for Phase I clinical trials from the China Center for Drug Evaluation (CDE). To assess the impact of this modification on the oncolytic efficacy of the virus in bladder cancer cells, M0-delADP, a control adenovirus, was used.

**Figure 1:**
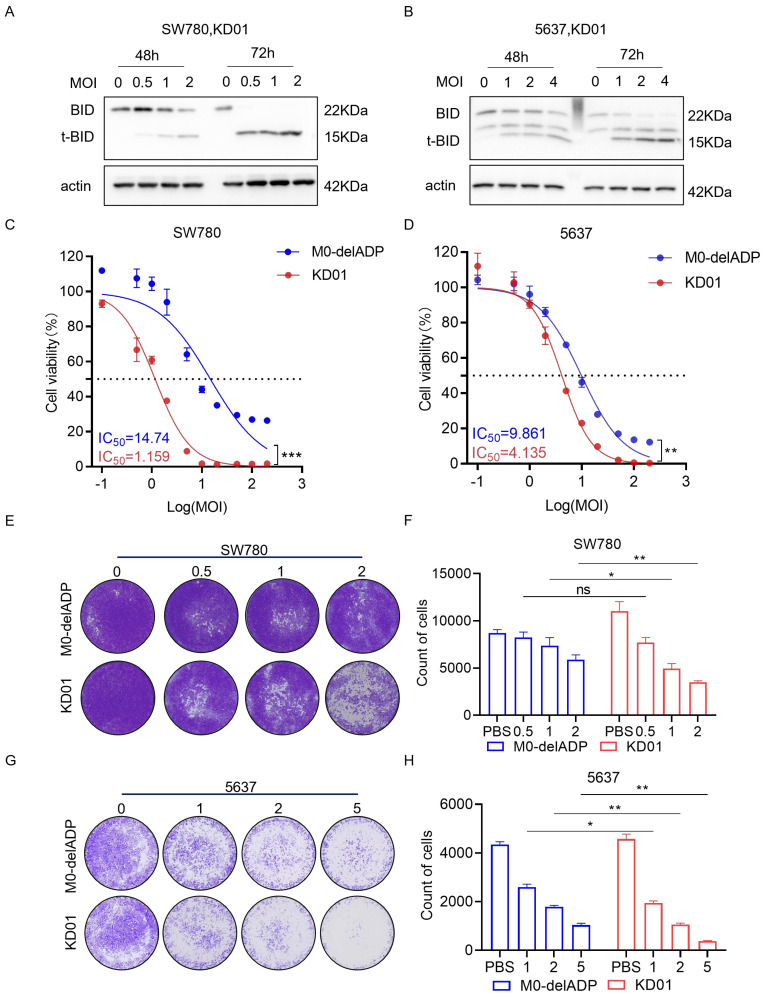
Killing effect of KD01 on bladder cancer cells in vitro. (**A**,**B**) The expression of tBID in SW780 cells (**A**) and 5637 cells (**B**) was detected by Western blot assay. (**C**,**D**) The inhibition rate of SW780 cells (**C**) and 5637 cells (**D**) by KD01 and M0-delADP for control was detected. Persistent infection for 72 h under the condition of multiplicity of infection (MOI) of 0.1, 0.5, 1, 2, 5, 10, 20, 50, 100, 200, the MOI was converted into a logarithm, the inhibition rate was converted into a percentage, and the IC_50_ value was calculated by a semi log fitting curve. Infective-free cell viability is determined by CCK8. ** *p* < 0.01, *** *p* < 0.005. (**E**–**H**) The inhibitory effect of KD01 on bladder cancer cells was determined by the plaque test in SW780 cells (**E**,**F**) and 5637 cells (**G**,**H**), Image J 1.54p was used to quantify plaque, and the data were derived from the average of three independent trials ± SD, ns indicated no significance, * *p* < 0.05.

KD01 demonstrated efficient replication in bladder cancer cell lines SW780 and 5637, with no significant difference in viral replication before and after the insertion of *tBID*, indicating that the modification did not alter the oncolytic properties of adenovirus (Figure S1A,B). Quantitative PCR and Western blot analysis confirmed a significant increase in tBID mRNA and protein levels following KD01 infection, with peak upregulation occurring between 48 and 72 h post-infection (Figure S1C,D and Figure 1A,B). Cytotoxicity assays performed at 72 h post-infection, when tBID protein levels were highest, and they showed significantly greater growth inhibition in SW780 and 5637 cells treated with KD01 compared to M0-delADP, with IC_50_ values of 1.159 µM for SW780 and 4.135 µM for 5637 cells at low MOI (Figure 1C,D). Colony formation assays further corroborated these findings, showing that KD01 had excellent ability to inhibit cell growth (Figure 1E–H). Additionally, morphological changes consistent with cytopathic effects, such as cell rounding and shrinkage, were observed following KD01 infection (Figure S2). Collectively, these results demonstrate that KD01 efficiently replicates and expresses in bladder cancer cells, induces high tBID expression, and exerts a potent cytotoxic effect, without compromising the oncolytic activity of the virus compared to the control adenovirus M0-delADP.

### 2.2. KD01 Infection Enhances Several Modes of Cell Death in Bladder Cancer Cells

**Figure 2:**
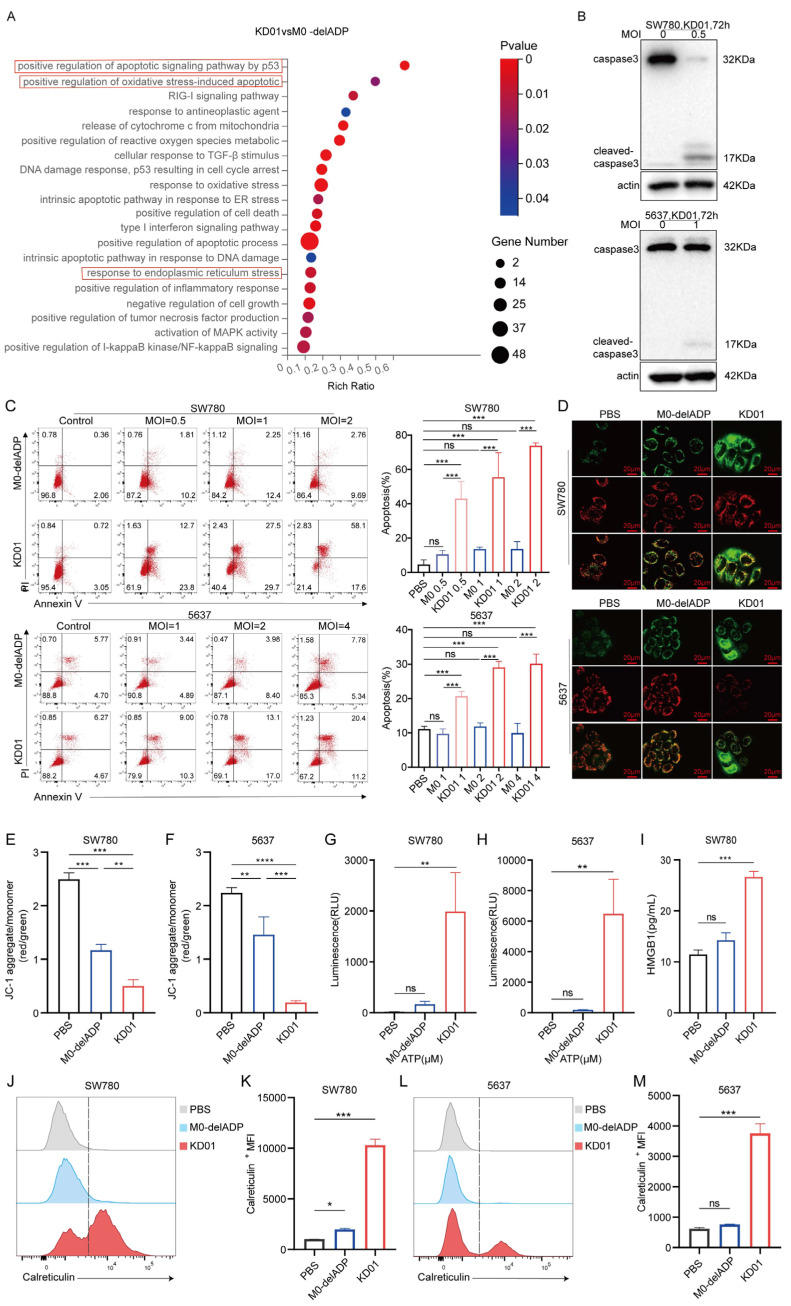
KD01 promotes mitochondrial pathway apoptosis and immunogenic cell death in bladder cancer cells (**A**) After SW780 cells were infected with M0-delADP/KD01 for 48 h, mRNA differences were analyzed by RNA-seq and differential functional pathways were enriched in the GO database. (**B**) The intact or cleaved caspase3 expression of SW780 and 5637 cells was detected by Western blot after 72 h treatment with KD01. (**C**) KD01/M0-delADP was incubated with SW780 and 5637 cells for 72 h, then Annexin V, PI-positive cells were quantitatively detected by flow cytometry. Annexin V and PI-positive cells are considered late apoptotic cells. The percentage of apoptotic cell populations is quantified. Data were obtained from three independent replicate experiments. (**D**–**F**) SW780 (MOI = 1) and 5637 (MOI = 2) cells were exposed to KD01/M0-delADP for 72 h, followed by JC-1 staining and photography, and the scale bar is 20 µm. (**G**,**H**) After 72 h incubation with KD01/M0-delADP, the content of ATP secreted by SW780 cells (MOI = 1) and 5637 cells (MOI = 2) was detected by the chemiluminescence method. Three valid experiments were performed. (**I**) The HMGB1 content secreted by SW780 cells was detected by ELISA. (**J**–**M**) The expression of calreticulin on the cell membrane of SW780 (MOI = 1) and 5637 cells (MOI = 2) after 72 h OVs treatment was measured by flow cytometry, single peak migration, and the data were represented by ΔMFI values. * *p* < 0.05, ** *p* < 0.01, *** *p* < 0.005, **** *p* < 0.0001, ns, *p* > 0.05.

To investigate the impact of oncolytic virus modification on alternative patterns of tumor cell death, we analyzed mRNA expression in KD01 or M0-delADP-infected SW780 cells through RNA sequencing. The results showed that the insertion of the pro-apoptotic gene tBID significantly enhanced the apoptotic process in tumor cells. Furthermore, KD01 infection also notably increased oxidative stress and endoplasmic reticulum stress (Figure 2A). These findings were confirmed through subsequent experiments. Western blot analysis demonstrated increased levels of cleaved caspase-3 and a corresponding decrease in full-length caspase-3 following KD01 infection (Figure 2B). Flow cytometry assays revealed that KD01 significantly elevated apoptosis levels in SW780 and 5637 cells in a dose-dependent manner, with a marked increase in late-stage apoptosis. Specifically, apoptosis rates increased by 60.1% in SW780 cells at MOI = 2 and by 20.2% in 5637 cells at MOI = 4 (Figure 2C). tBID induces apoptosis through the endogenous apoptotic pathway by promoting Bak and Bax activation, which perforate the mitochondrial membrane, leading to mitochondrial outer membrane permeabilization (MOMP). Mitochondria play a critical role in cellular stress signaling and are central to various cell death processes [22]. We assessed MOMP using JC-1, which revealed a significant decrease in red fluorescence (JC-1 polymer) and an increase in green fluorescence (JC-1 monomer), indicating reduced mitochondrial membrane potential following KD01 infection. (Figure 2D–F). These results suggest that KD01 induces apoptosis in bladder cancer cells via the mitochondrial pathway.

Moreover, immunogenic cell death (ICD) plays a crucial role in tumor immunotherapy. Enhanced oxidative stress and endoplasmic reticulum stress are known to promote ICD [23], suggesting that KD01 may also facilitate this process. To investigate this, we evaluated the release of damage-associated molecular patterns (DAMPs) in KD01-infected cells. The secretion of adenosine triphosphate (ATP) in SW780 and 5637 cells, and release of high mobility group protein B1 (HMGB1) in SW780 cells at 72 h, were notably elevated (Figure 2G–I). Additionally, KD01 significantly increased the surface expression of calreticulin in both SW780 and 5637 cells at 72 h post-infection (Figure 2J–M). These results confirm that KD01 consistently induces DAMP release in bladder cancer cell lines.

Taken together, these findings indicated that KD01 not only induces apoptosis through the mitochondrial pathway but also enhances the immunogenicity of bladder cancer cells by promoting DAMP release, suggesting its potential to stimulate robust anti-tumor immune responses.

### 2.3. Cisplatin and KD01 Combination Increased Tumor Cell Apoptosis and Immunogenicity

It has been clarified that KD01 single-agent use kills bladder cancer cells by promoting apoptosis and enhancing the immunogenicity of cancer cells. In view of the reported immunomodulatory and apoptosis-promoting effects of cisplatin, we explored the effect of combining KD01 with cisplatin to provide data supporting the efficacy of sensitizing oncolytic viruses while using cisplatin in small doses for systemic chemotherapy.

**Figure 3:**
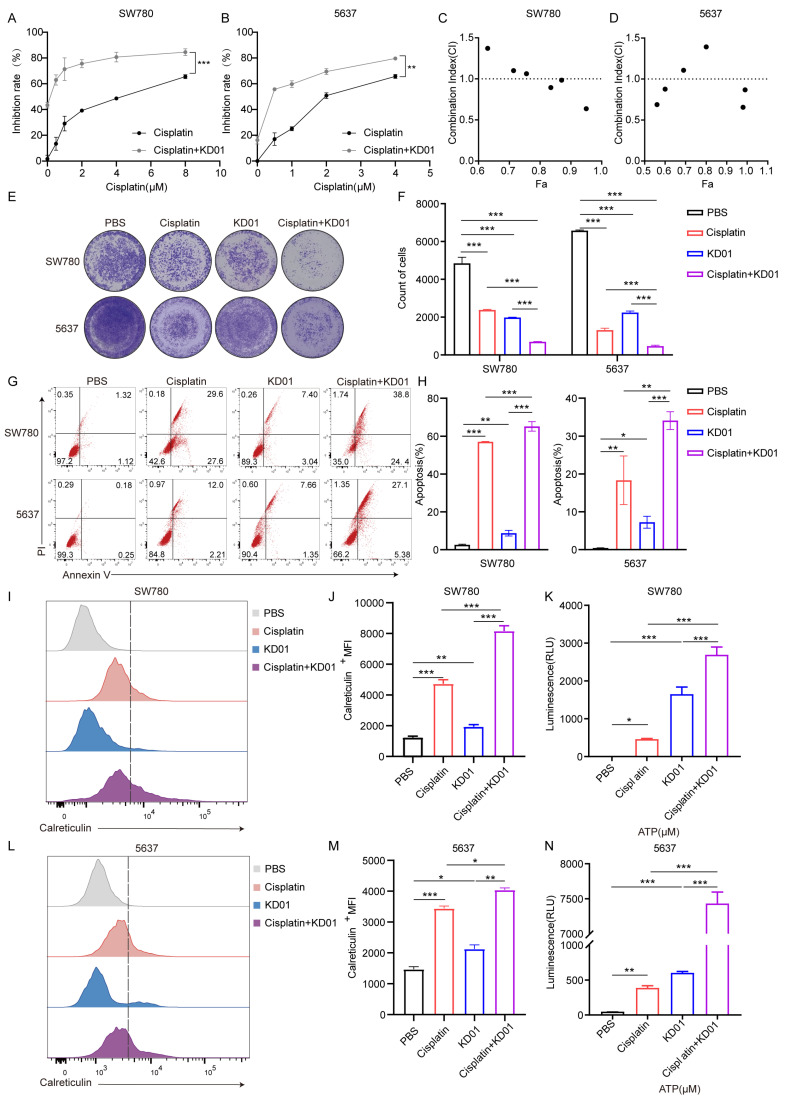
KD01 and cisplatin synergistically promote apoptosis and ICD in bladder cancer cells (**A**,**B**). Cells were treated with a fixed concentration of KD01 and different doses of cisplatin: for SW780 cells, the doses were 0.5 µM, 1 µM, 2 µM, 4 µM, and 8 µM; for 5637 cells, the doses were 0.5 µM, 1 µM, 2 µM, and 4 µM. After 72 h, cell viability was assessed using CCK8, and the inhibition rates were calculated. Data from three independent experiments were presented as mean ± SD. (**C**,**D**) After the inhibition rate was measured by CCK8, the synergistic index (CI) was calculated by Compusyn. CI < 1 indicated that the two drugs had synergistic effects. (**E**,**F**) KD01 was co-cultured with SW780 cells (MOI = 0.5) or 5637 cells (MOI = 2) for 72 h, simultaneously treating them with cisplatin, SW780 cells (4 µM) and 5637 cells (2 µM). The inhibitory effect of KD01 combined with cisplatin on bladder cancer cells was determined by the plaque test in SW780 cells and 5637 cells, Image J 1.54p was used to quantify plaque. All experiments were repeated three times. (**G**,**H**) After 72 h incubation of KD01 and cisplatin with SW780 cells (MOI = 0.5, 4 µM) or 5637 cells (MOI = 2, 2 µm), flow cytometry was used to detect Annexin V and PI-positive cells. The percentage of apoptotic cell populations is quantified. The data comes from three experiments. (**I**,**J**) KD01 and cisplatin were co-incubated with SW780 cells (MOI = 0.5, 4 µM) for 72 h, and then flow cytometry was used to detect calreticulin expression on the cell membrane. Data were represented by ΔMFI values. (**K**) SW780 cells were incubated with KD01 with MOI = 0.5 and cisplatin with a concentration of 4 µM for 72 h, and the content of ATP secreted by the cells was detected by the chemiluminescence method. The data were collected from three independent experiments. (**L**–**N**) KD01 and cisplatin were simultaneously treated with 5637 cells (MOI = 2, 2 µM) for 72 h, and then the expression of calreticulin on the cell membrane and the content of ATP secreted by 5637 cells were detected using the same experimental methods. * *p* < 0.05, ** *p* < 0.01, *** *p* < 0.005.

Initial experiments determined the IC_50_ values of cisplatin for SW780 and 5637 cells as 9.301 μM and 1.602 μM, respectively (Figure S3). Combination treatment with KD01 and cisplatin significantly increased the inhibition rate of bladder cancer cells compared to cisplatin alone, with a 32.12% increase in SW780 cells at 4 μM and a 34.54% increase in 5637 cells at 1μM cisplatin (Figure 3A,B). Drug combination index analysis indicated a synergistic effect at high inhibition rates (Figure 3C,D). Colony formation assays further confirmed that combined treatment markedly reduced the number of cell colonies (Figure 3E,F). Apoptosis analysis revealed that combination therapy significantly increased late-stage apoptosis compared to monotherapy (Figure 3G,H). Given the known ability of cisplatin to enhance ICD in certain cancer species and the ability of KD01 to enhance immunogenicity, we investigated their combined effects on ICD markers. In SW780 cells, combination treatment significantly upregulated surface calreticulin expression and elevated ATP and HMGB1 secretion compared to either agent alone (Figure 3I–K and Figure S4). Similar effects were observed in 5637 cells with increased calreticulin membrane expression and extracellular ATP levels (Figure 3L–N).

These findings demonstrate that cisplatin not only sensitizes bladder cancer cells to KD01 but also enhances their immunogenicity, suggesting that the combination of KD01 and cisplatin has a dual benefit in terms of improving therapeutic efficacy and stimulating anti-tumor immunity.

### 2.4. KD01 Inhibits Tumor Growth and Enhances Apoptosis In Vivo in Bladder Cancer Models

To assess the in vivo anti-tumor effects of KD01, SW780 cells (1 × 10^7^) were inoculated into mice, which were then treated with PBS, M0-delADP, or KD01 via intra-tumoral injection. Mouse-derived bladder cancer cells (MB49) are inherently insensitive to our oncolytic adenovirus (Figure S5); therefore, we selected BALB/c-nude mice to create a SW780 tumor-bearing mice for the experiment.

**Figure 4:**
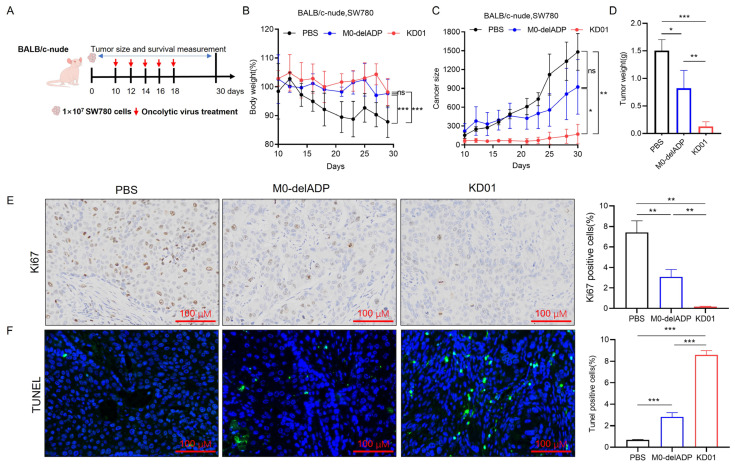
Therapeutic effect of KD01 on SW780 tumor-bearing mice. (**A**) Diagram of mouse experimental. (**B**) The body weight curve of mice during KD01/M0-delADP treatment. (**C**) Record of tumor growth in each group of mice. (**D**) Comparison of tumor weights from different groups of SW780 cells in the D30 xenografts. (**E**,**F**) After euthanizing each group of mice, tumor tissues were immediately harvested, and immunohistochemical staining was used to detect the expressions of Ki67 and TUNEL in the tissues, the scale bar is 100 µm. Image J 1.54p was employed to analyze the proportion of positive cells, and the data are presented as mean ± SD.* *p* < 0.05, ** *p* < 0.01, *** *p* < 0.005, ns, *p* > 0.05.

Tumor growth was monitored for 30 days post-inoculation (Figure 4A). KD01 and M0-delADP treatments alleviated weight loss in mice compared to PBS (Figure 4B). After five doses, on day 29, the average tumor volume in the PBS group increased by 861.5% compared to day 10 (before treatment), in the M0-delADP group increased by 316.43%, while in the KD01 group increased by 159.64%, indicating significant tumor growth inhibition with KD01 (Figure 4C). At the end of observation, the mean tumor weight of the M0-delADP group was 1.83-fold lower than that of the PBS group, and that of the KD01 group was 11.83-fold lower than that of the PBS group (Figure 4D). Immunohistochemical experiments revealed that after M0-delADP/KD01 treatment, the expression of nuclear division and proliferation-related protein (Ki67) in tumor tissue decreased, while the level of terminal deoxynucleotidyl transferase dUTP nick end labeling (TUNEL) increased, indicating a weakened proliferative ability and increased apoptosis levels in tumor tissue after treatment (Figure 4E,F). These results confirm that KD01 effectively inhibits tumor growth in vivo.

### 2.5. The Safety of KD01 Was Evaluated Using a Syrian Hamster Bladder Perfusion Model

**Figure 5:**
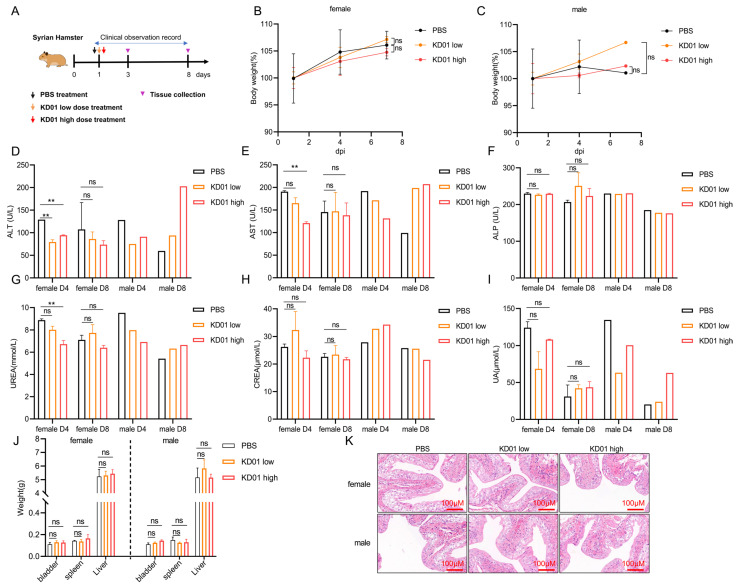
KD01 bladder perfusion safety test. (**A**) Syrian hamster model of bladder perfusion experiment setup. (**B**,**C**) Male/female Syrian hamster body weight change curve. (**D**–**F**) The venous blood of hamsters was collected for routine blood and blood biochemical tests on day 3 (D4) and day 7 (D8) after administration, respectively, and the levels of alanine aminotransferase (ALT), aspartate aminotransferase (AST) and alkaline phosphatase (ALP) were recorded. The data were expressed as mean ± SD. There were two females (*n* = 2) and one male (*n* = 1), but due to the limited number of male individuals, statistical difference analysis could not be performed. (**G**–**I**) Serum UREA nitrogen (UREA), serum creatinine (CREA), and serum uric acid (UA) levels in venous blood of hamsters were measured at D4 and D8, as mean ± SD, for female (*n* = 2) and male (*n* = 1). (**J**) The weight of the organs of each group was compared after killing the hamsters, and the data were expressed as mean ± SD, female (*n* = 4) and male (*n* = 2). (**K**) Pathological examination of bladder tissue in each group was performed to observe the pathological injury of bladder tissue; the scale bar is 100 µm. ** *p* < 0.01, ns, *p* > 0.05.

To evaluate the safety of KD01 bladder instillation, we conducted a toxicity study using 18 Syrian hamsters, which were divided into three groups: PBS control, low-dose, and high-dose. Following a single bladder perfusion, the animals were monitored for seven days (Figure 5A). Clinical observations, body weight measurements, blood tests, and organ weight assessments were performed throughout the study. No fatalities or abnormalities were observed during clinical examinations. All groups demonstrated slight weight gain, with no significant differences between groups (Figure 5B,C). Blood biochemistry tests on days 4 and 8 showed no significant changes in alkaline phosphatase (ALP), serum creatinine (CREA), or serum uric acid (UA) levels in the treatment groups. There was a transient decrease in alanine aminotransferase (ALT), aspartate aminotransferase (AST), and urea (UF) levels, which returned to normal by day 8, suggesting no significant liver or kidney dysfunction (Figure 5D–I). Organ weight analysis revealed no substantial changes in the bladder, spleen, or liver (Figure 5J). Pathological examination of bladder tissues on day 8 revealed intact epithelium with no signs of cytological abnormalities, inflammation, or damage in the KD01-treated groups compared to controls (Figure 5K). These findings indicate that KD01 bladder instillation is well-tolerated and supports its clinical potential for the treatment of bladder cancer.

## 3. Discussion

In recent years, the oncolytic virus, as a new cancer therapeutic agent, has demonstrated its advantages in treating bladder cancer, with intravesical delivery allowing easy access to the disease site while bypassing the systemic antiviral humoral response to ensure adequate drug delivery [24,25]. Based on the efficacy of oncolytic viruses against bladder cancer, in this article, we found that the modified oncolytic adenovirus KD01 exhibited an effective killing effect on bladder cancer cells both in vivo and in vitro, which was more pronounced compared with the unmodified oncolytic adenovirus. Oncolytic viruses destroy tumor cells and activate immune responses, replicating within these cells to cause lysis and release new particles [26]. This process induces cell death and the release of signaling molecules, including PAMPs and DAMPs, which can restore the immune response against tumors [27,28].

A major problem facing oncolytic viruses currently lies in the heterogeneity of tumor cells leading to their inefficient replication in specific tumor cells. In some cases, viral infection induces autophagy, while infected cells die before virus release, which limits further lysis of the virus [29,30,31]. Elevating the expression of apoptotic genes may be the key to addressing this problem; however, this may raise concerns about the reduced ability of the virus to replicate within the cell. Our study showed that the expression of the tBID gene did not compromise the replication capability of KD01 within bladder tumor cells and the modification of KD01 did not affect tumor cell death due to viral replication; however, tBID induced the formation of pores in the mitochondrial membrane, enhancing its permeability and elevating the apoptotic rate of the bladder cancer cells, particularly at the late stage of apoptosis. The increase in tBID expression accelerated tumor cell death, suggesting that our modification improved the efficiency of oncolytic viruses.

Moreover, the RNA-seq results in our study indicate that KD01 infection intensifies endoplasmic reticulum stress and oxidative stress in tumor cells—two biological processes capable of inducing immunogenic cell death—while also amplifying the inflammatory response of the tumor cells [32]. Additional investigations have demonstrated that KD01 induces upregulation of calreticulin expression on the cellular membrane and facilitates the extrusion of ATP and HMGB1 from the cells. These findings substantiate that KD01 infection elicits ICD in bladder cancer cells, a process characterized by activating an immune response to the antigens of deceased cells [33,34]. This indicates that KD01 directly kills bladder cancer cells and enhances their immunogenicity, potentially improving the anti-tumor immunity in vivo and thereby killing tumor cells through a dual mechanism.

We found that the combination of KD01 and cisplatin enhanced the sensitivity of bladder cancer cells to KD01, and the combination treatment further promoted apoptosis, and there was a synergistic effect between the two at high inhibition rates, suggesting that the combination could enhance the anti-tumor effect. We detected that the combination of KD01 and cisplatin also promoted ICD in bladder cancer cells. Therefore, we believe that cisplatin can enhance the efficacy of KD01, and that it is also helpful in terms of enhancing the anti-tumor immunity, which adds an additional mode of use to the clinical use of KD01. Currently there are a variety of choices of chemotherapeutic and immunotherapeutic agents used for the treatment of bladder cancer, and when used clinically, they are not only limited to the combination with cisplatin. The ideal way of administration of KD01 for NMIBC patients is bladder perfusion therapy, which can easily reach the site of the tumor and adequately deliver the drug. The therapeutic effect of KD01 bladder perfusion is limited for patients with muscle invasive bladder cancer. The enhanced anti-tumor effect of the combination of KD01 and cisplatin suggests that KD01 can be used in combination with other chemotherapeutic drugs in our clinical practice, and that the combination of KD01 bladder perfusion with systemic chemotherapeutic drugs adjuvant treatment, in the enhancement of oncolytic virus tumor lysis effect at the same time, perhaps can inhibit the metastasis of bladder cancer and other progress. For the treatment of patients with muscle invasive bladder cancer, this provides a new idea, and the future is a therapeutic modality that can be explored.

To assess the in vivo killing effect of KD01, we selected BALB/c nude mice to establish the SW780 bladder cancer model for our experiments, as mouse tumor cells are not an ideal model for verifying the efficacy of oncolytic adenovirus [35,36]. After intra-tumoral administration, we found that unmodified adenovirus had a slight inhibitory effect on tumor growth. In contrast, modified KD01 had a more significant inhibitory effect on tumor growth, inhibited tumor proliferation, and promoted apoptosis of tumor cells in vivo. After treatment of Syrian hamsters with KD01 bladder perfusion, no abnormalities were found in their clinical examination, body weight, and blood tests, and there were no signs of inflammation or injury in the bladder, indicating that KD01 bladder perfusion was well tolerated and had a favorable safety profile. Supported by the data in this post, the clinical trial for KD01 in treating bladder cancer has completed review and has been approved for IND, with a clinical trial to be conducted soon (Clinical Trial Acceptance Number: MR-42-24-038517).

The shortcomings of this study include the following: The mechanism of KD01 oncolytic needs to be refined; we mainly found that KD01 causes bladder cancer cells to undergo apoptosis and ICD; whether KD01 also promotes other modes of cell death in tumor cells was not fully explored; the mechanism of KD01 oncolytic may be elucidated in subsequent studies. In addition, the RNA sequencing results suggest that KD01 infection enhances the inflammatory response of bladder cancer cells, and whether the enhanced inflammatory response has a positive or negative inhibitory role against the tumor immune response in vivo has not been clarified.

In conclusion, this study demonstrates that KD01, a tumor-specific oncolytic adenovirus carrying tBID, promotes apoptosis and induces ICD in bladder cancer cells, killing them through dual pathways. Combination with the chemotherapeutic agent cisplatin enhances the efficacy of KD01, providing data to support the efficacy of sensitizing oncolytic viruses in conjunction with systemic chemotherapy using cisplatin in small doses. Safety assessment in healthy Syrian hamsters showed that KD01 was well tolerated by bladder perfusion. This provides a new option for the treatment of bladder cancer patients, as well as new evidence and reference for the clinical use and efficacy of KD01, and it supports pharmacological and safety data for conducting clinical trials of KD01 bladder instillation therapy in bladder cancer.

## 4. Materials and Methods

### 4.1. Cell Lines, Viruses, and Compounds

Human bladder metastatic carcinoma cells (SW780) and mouse bladder carcinoma cells (MB49) were obtained from Wuhan Punosai Biotechnology Co. Ltd. (Wuhan, China), while human bladder carcinoma cells (5637) were purchased from Applied Biological Materials Inc (ABM, Richmond, BC, Canada). SW780 and MB49 cells were cultured in DMEM supplemented with 15% and 10% fetal bovine serum (FBS), respectively, and 10,000 U/mL Penicillin-Streptomycin Solution. A total of 5637 cells were cultured in RPMI 1640 medium supplemented with 10% FBS and 10,000 U/mL Penicillin-Streptomycin Solution. All cell cultures were maintained in an incubator at 37 °C with 5% CO_2_.

Two adenoviruses were used in this study: M0-delADP, an E1A CR2 and E3 ADP deletion adenovirus mutant, and KD01, which contains the pro-apoptotic tBID gene inserted into the ADP region of M0-delADP. Both M0-delADP and KD01 were provided by Wuhan Kadwise Biotechnology Co., Ltd. (Wuhan, China). Cisplatin (HY-17394) was purchased from MedChemExpress (MCE, Shanghai, China), dissolved in pure water to prepare a 2 mM stock solution, and stored at −80 °C until use.

### 4.2. Real-Time qPCR (RT-qPCR) Analysis

Total RNA or DNA was extracted from cultured cells using the FastPure Cell/Tissue Total RNA Isolation Kit V2/Tissue DNA Kit D3396 kit according to the manufacturer’s instructions. RNA was reverse transcribed to cDNA using the ABScript Neo RT Master Mix for qPCR with gDNA Remover kit and stored at −20 °C or used directly for qPCR. SYBR Green-based qPCR was performed using 2× Universal SYBR Green Fast qPCR Mix (ABclonal, Wuhan, China). The primer sequences used were as follows. *BID*, forward: 5′-ATGGACCGTAGCATCCCTCC-3′, reverse: 5′-GTAGGTGCGTAGGTTCTGGT-3′; Fiber, forward: 5′-ACTATATGGACAACGTCAACCCATT-3′, reverse: 5′-ACCTTCTGAGGCACCTGGATGT-3′; *GAPDH*, forward: 5′-GGAGCGAGATCCCTCCAAAAT-3′, reverse: 5′-GGCTGTTGTCATACTTCTCATGG-3′. Data were normalized to *GAPDH* when calculating the relative expression of the target gene. qPCR was performed using a CFX96 real-time PCR system (Bio-Rad, Hercules, CA, USA). Data were analyzed by the Bio-Rad CFX Manager using the normalized expression pattern (2^−ΔΔCq^). Three measurements were performed.

### 4.3. Western Blot

Intact cell contents were obtained using cold RIPA lysates containing cocktail, phosphorylated protease inhibitor A solution and B solution, using the BCA^TM^ protein assay kit assay the protein concentration. The proteins were separated by 10% SDS-PAGE gel. The separated proteins were transferred onto a 0.45 µm PVDF membrane, which was blocked with a protein-free rapid-blocking solution for 1 h. Then, they were incubated with the primary antibodies related to the target proteins overnight. The following antibodies were used: BID Antibody (CST, #2002, Shanghai, China), Caspase-3 Antibody (Proteintech, 82202-1, Wuhan, China), Anti-beta Actin (ab6276, abcam, Shanghai, China). After incubation, continue to incubate the corresponding secondary antibodies. After incubation, corresponding secondary antibodies. Finally, the proteins were visualized using the BeyoECL Plus chemiluminescence kit (Beyotime, Shanghai, China). Development results were analyzed in fixed grayscale using Image-pro-plus Version 6.0, and the analyzed data were normalized to β-actin.

### 4.4. Crystalline Violet Staining Assay

Cells were inoculated in 96-well (7 × 10^3^/well) dishes overnight, infected with viral particles at different MOIs, or treated with cisplatin. After 72 h of inoculation, the supernatant containing viral particles or cisplatin was removed. Cells were fixed and then stained with a crystal violet staining solution, leaving them to dry for photographic recording. The number of clones was counted using Image J 1.54p.

### 4.5. Apoptosis and Flow Cytometry Analysis

Cells were inoculated in 6-well dishes (2 × 10^5^/well) for overnight growth, co-incubated by adding M0-delADP/KD01, and treated with different concentrations of cisplatin in the viral solution when the drugs were used in combination, and the cells were collected after 72 h of co-incubation, the cells were assayed for apoptosis using the FITC Annexin V Apoptosis Detection Kit I (BD Biosciences, Franklin Lakes, NJ, USA) for apoptosis detection. Samples for calreticulin assay were prepared as above and stained using calreticulin antibody (CST, #19780, Shanghai, China) and assayed on the machine. Data using a FACSCalibur flow cytometer (BD Biosciences, Franklin Lakes, NJ, USA) were analyzed.

### 4.6. RNA Seq Analysis

During the sample processing stage, SW780 cells were inoculated into 6-well (2 × 10^5^/well) culture dishes overnight, and 0.5 MOI of virus was added. After co-incubation for 48 h, the supernatant was aspirated, trypsin digestion was performed to collect the cells in cell freezing tubes, and the supernatant was aspirated and placed in liquid nitrogen for storage. Transcriptomics sequencing and analysis were assigned to BGI Genomics.

### 4.7. JC-1 Mitochondrial Membrane Potential Assay

Different viral particle treatments were added to the cells inoculated in confocal Petri dishes, and the cellular mitochondrial membrane potential was detected using the JC-1 (Beyotime Biotechnology Co., Shanghai, China) kit after 72 h of infection. The cells in the dish were washed once with PBS, 1 mL of cell complete medium was added, then 1 mL of prepared JC-1 working solution was added, and the cells were incubated at 37 °C. The supernatant was aspirated, washed twice, and 2 mL of cell culture medium was added, observing the cells under a laser confocal microscope.

### 4.8. Enzyme-Linked Immunosorbent Assay (ELISA)

SW780 cells were treated with KD01/M0-delADP with MOI = 1 when combined with cisplatin. A total of 2 µM of cisplatin was added to the viral solution simultaneously. After 72 h, the supernatant was stored at −80 °C in a refrigerator, and it was melted on ice before the assay. HMGB1 levels were determined using the Human High Mobility Group Protein B1 ELISA Kit (Sangon Biotech, D711210, Shanghai, China).

### 4.9. Immunogenic Cell Death Detection Assay

Cells were inoculated with virus particles of different MOIs or treated by adding cisplatin. The supernatants in the well plates were collected after 72 h of treatment, and then ATP levels in the supernatants of the cell culture plates were detected by using the ATP Assay Kit (Beyotime). The remaining supernatants were stored at −80 °C and HMGB1 levels were measured using ELISA. Calreticulin assay was analyzed using flow cytometry as previously described.

### 4.10. Cell Growth Inhibition Assay with CI Index Analysis

The half inhibitory concentration (IC_50_) of KD01 and the cell inhibitory assay in combination with cisplatin were determined using the CCK8 assay (Vazyme, Nanjing, China). Various multiplicities of infection (MOI) of M0-delADP or KD01, diluted in complete cell culture medium, were co-incubated with SW780 or 5637 cells seeded in 96-well plates for 72 h in a 37 °C incubator, with a blank cell negative control.

For the drug combination inhibition assay, KD01 at MOI = 1 was added to SW780 cells, and KD01 at MOI = 2 was added to 5637 cells. Cisplatin treatment was applied at various concentrations, and the cells were incubated for 72 h. Following incubation, the supernatant was discarded, and CCK8 working solution was added. Cell viability was measured using a microplate reader, which reflects the amount of reduced methazolene dye. The inhibition rate and IC_50_ values were calculated using GraphPad Prism 8.0. The combination index (CI) was calculated using Compusyn software 1.0. The experiment was repeated three times with at least three parallel measurements per experiment to obtain mean and standard deviation (SD) values.

### 4.11. In Vivo Therapeutic Effect Evaluation Experiment

The study used 6–8-week-old female BALB/c-nude mice, purchased from Mouse Lai Bao (Wuhan) Biotechnology Co., Ltd. (Wuhan, China). A total of 1 × 10^7^ SW780 cells were subcutaneously inoculated into the right axilla of the animals. After 10 days, the tumor-bearing animals were screened for subsequent experiments, with the researchers blinded to the group assignments. The mice were randomly put into three groups: PBS control, M0-delADP treatment, and KD01 treatment, with three animals in each group. Intratumoral injections of 50 μL of oncolytic viruses (OVs) or PBS were administered every two days starting from the 10th day of inoculation, with a viral dose of 1 × 10^8^ PFU per injection, for five treatments.

From the day of treatment, weight was recorded, and tumor volume was measured using vernier calipers (volume = length × width × width × 0.5). The animals were continuously observed for 30 days following cell inoculation. At the end of the observation period, tumors were dissected, weighed, and fixed in 4% paraformaldehyde for further analysis. The experimental animals were housed in the pathogen-free animal facility at Tongji Medical College.

### 4.12. Safety Evaluation Test

The safety evaluation of KD01 in vivo was conducted using 18 Syrian hamsters (12 females and 6 males) aged 6–8 weeks, purchased from Beijing Viton Lihua Laboratory Animal Co. Ltd. (Beijing, China). The hamsters’ body weights were measured, and they were divided into three groups: the control group, the low-dose group, and the high-dose group, with 6 hamsters (4 females and 2 males) in each group. The bladder was perfused with a single dose of 100 μL of PBS in the control group, 100 μL of virus solution at 8.6 × 10^9^ VP per pup in the low-dose group, and 100 μL of virus solution at 2.6 × 10^10^ VP per pup in the high-dose group.

On D1, D4, andD8 after administration, 3 animals per group (2 females and 1 male) were euthanized for analysis. Throughout the testing period, clinical observations, body weight measurements, clinicopathological tests (blood routine, blood biochemistry), and organ dissection for weight assessment were performed.

### 4.13. H&E Pathological Experiment

Mouse tissues used for pathological examination were fixed in 4% paraformaldehyde fixative for at least 24 h. Subsequently, they were processed by paraffin embedding and randomly sliced, which were deparaffinized by xylene and then hydrated by immersion in ethanol. Finally, the tissue sections were stained by using hematoxylin-eosin and then dried for observation and recording by using a light microscope.

### 4.14. Immunohistochemical Assay with TUNEL Staining

Tissue sections were dewaxed with xylene and rehydrated in ethanol. Antigen retrieval was performed by autoclaving the sections in the citrate buffer. Endogenous peroxidase activity was then blocked by 0.3% H_2_O_2_ in the dark after washing three times with PBS, blocked with 5% BSA, and overnight incubation with Ki67 primary antibody. After washing and incubation with a biotinylated secondary antibody (Abcam, Shanghai, China), the sections were stained with DAB and visualized with microscope.

For TUNEL staining, the tissue sections were rehydrated in ethanol, permeabilized with proteinase K, and equilibrated in Equilibration Buffer for 30 min. Terminal deoxyribonucleotidyl transferase (TdT)-labeling buffer was added, followed by PBS washes. Then, it was dyed with DAPI and observed. Ki67 histochemistry and TUNEL positivity were quantified using Image J 1.54p.

### 4.15. Statistical Analysis

GraphPad Prism (version 8.0; GraphPad Software, La Jolla, CA, USA) was used for analysis, employing an unpaired Student’s *t*-test or one-way ANOVA for statistical significance, with Tukey’s multiple comparison test used when comparing multiple groups. The results are presented as means and SD from three independent experiments, with a *p*-value of less than 0.05 considered statistically significant.

## 5. Conclusions

In conclusion, KD01 demonstrated potent anti-tumor efficacy, showing significant therapeutic potential for bladder cancer. When combined with cisplatin, KD01 sensitized bladder cancer cells, enhancing its efficacy by promoting apoptosis and immunogenic cell death, which may further bolster anti-tumor immunity. A preclinical safety assessment of KD01 bladder perfusion therapy revealed no significant toxicity in Syrian hamsters and no adverse effects on organ function or bladder tissue integrity. These results support the potential of KD01 as a promising therapeutic option for bladder cancer, laying a strong foundation for its continued clinical evaluation and offering new treatment possibilities for bladder cancer patients.

## Data Availability

Data of this study are included in the article.

## Supplementary Materials

The following supporting information can be downloaded at: https://www.mdpi.com/article/10.3390/ph18040511/s1, Figure S1: KD01 was effectively replicated and expressed in bladder cancer cells; Figure S2: KD01’s killing of bladder cancer cells; Figure S3: Effective killing dose of cisplatin in bladder cancer cells; Figure S4: KD01 in combination with cisplatin promoted the extracellular release of HMGB1; Figure S5: KD01 killing effects on MB49 cells.
