# Supplementary material for: Preclinical Efficacy and Safety of an Oncolytic Adenovirus KD01 for the Treatment of Bladder Cancer
Source: Pharmaceuticals (Basel). 2025 Mar 31;18(4):511. doi: 10.3390/ph18040511 (PMC12030491; doi:10.3390/ph18040511)
Supplement: Supplementary file 1 [file pharmaceuticals-18-00511-s001.zip › pharmaceuticals-3528485-supplementary.pdf]

Figure. S1

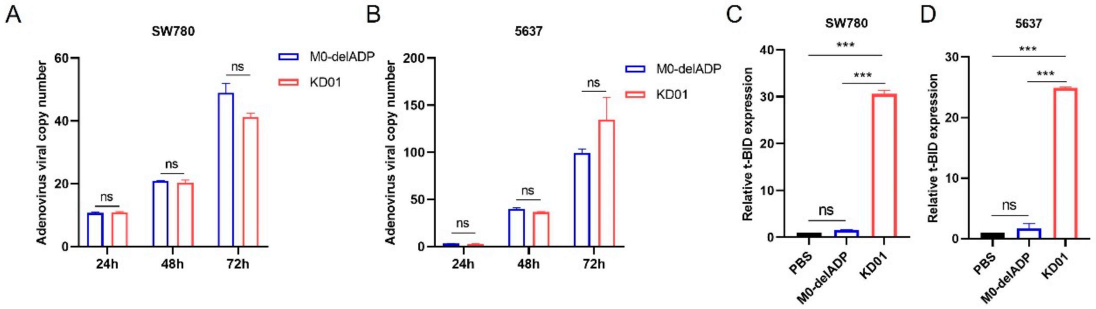

Figure.S1 KD01 was effectively replicated and expressed in bladder cancer cells. (A, B) The whole-cell DNA was collected after 24h, 48h, 72h of KD01 infection in SW780 cells (MOI=0.5) and 5637 cells (MOI=2), and the Fiber gene was quantitatively detected by qPCR. (C, D) Total RNA was extracted 72h after KD01 infected SW780 cells (MOI=1) or 5637 cells (MOI=2) and tBID expression was detected by RT-qPCR.

Figure. S2

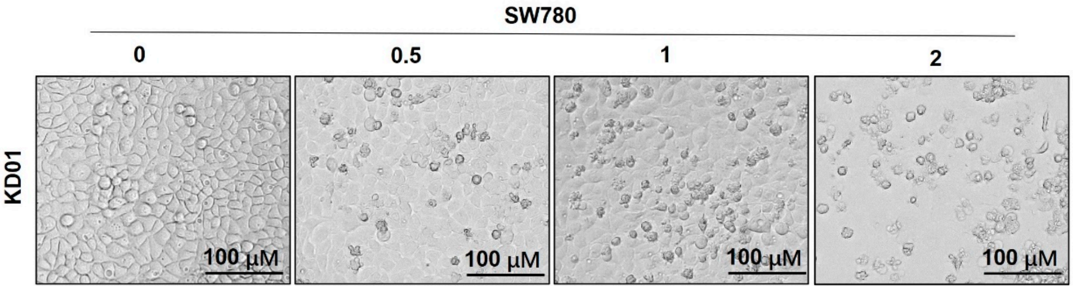

Figure.S2 Killing of bladder cancer cells by KD01. The CPE of SW780 cells was observed and recorded by optical microscope after KD01 infection 72h.

Figure. S3

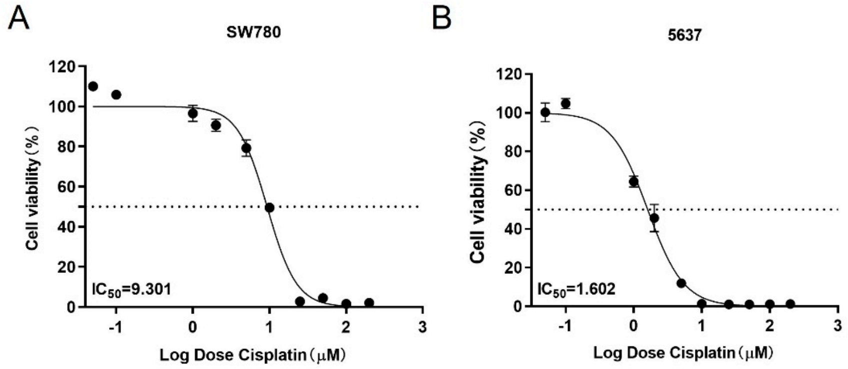

Figure.S3 Effective killing dose of cisplatin in bladder cancer cells. (A, B) The inhibitory rate of cisplatin on SW780 cells (A) and 5637 cells (B) was detected by CCK8. When the concentration was 0.05, 0.1, 1, 2, 5, 10, 25, 50, 100, and 200 for 72 h, the concentration was converted into pairs, the inhibition rate was converted into a percentage, and the IC_{50} value was calculated using a semi-logarithmic fitting curve.

Figure. S4

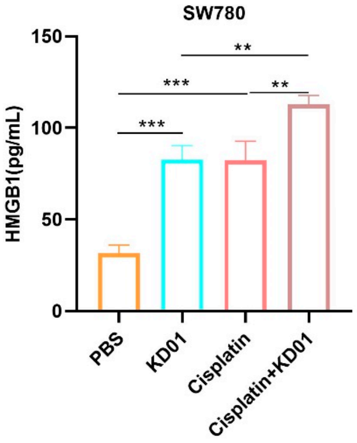

Figure.S4 Combination of KD01 and cisplatin promotes HMGB1 release from SW780 cells. The level of HMGB1 in cell supernatants was measured using an ELISA kit.

Figure. S5

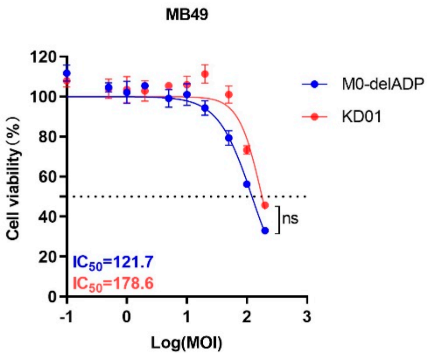

Figure.S5 KD01 killing effects on MB49 cells. The inhibitory rates of KD01 and M0-delADP on MB49 cells were detected. In the condition of multiplicity of infection (MOI) of 0.1, 0.5, 1, 2, 5, 10, 20, 50, 100, and 200 for 72 hours, CCK8 detected the viability of non-infected cells as a control.
